# Location, location, location: Nuclear pore complexes tether flowering loci to nuclear envelope to boost gene expression

**DOI:** 10.1093/plcell/koad282

**Published:** 2023-11-07

**Authors:** Carlisle Bascom

**Affiliations:** Assistant Features Editor, The Plant Cell, American Society of Plant Biologists; Natural Resources and the Environment Department, University of New Hampshire, Durham, NH 03824, USA

In real estate, the three most important factors are location, location, location. But is the same true for a gene locus in the nucleus? Well, research in mammalian cells has discovered that nuclear pore complexes, in addition to regulating transport into and out of the nucleus, affect gene expression by physically tethering the locus to the nuclear envelope (D’Angelo 2018; Brickner et al. 2019). The extent to which plant cells similarly regulate gene expression is understudied. Previous work found that loci of *FLOWERING LOCUS C* (*FLC*) shifted to the nuclear periphery in response to temperature, though the exact mechanism was unclear (Rosa et al. 2013). In this issue of *The Plant Cell*, **Penghui Huang and colleagues** found that a specific nuclear pore subcomplex, the Y-complex, regulates the expression of *FLC* in *Arabidopsis* by physically moving the locus closer to the periphery of the nucleus (Huang et al. 2023) (see Fig. 1).

**Figure 1.**
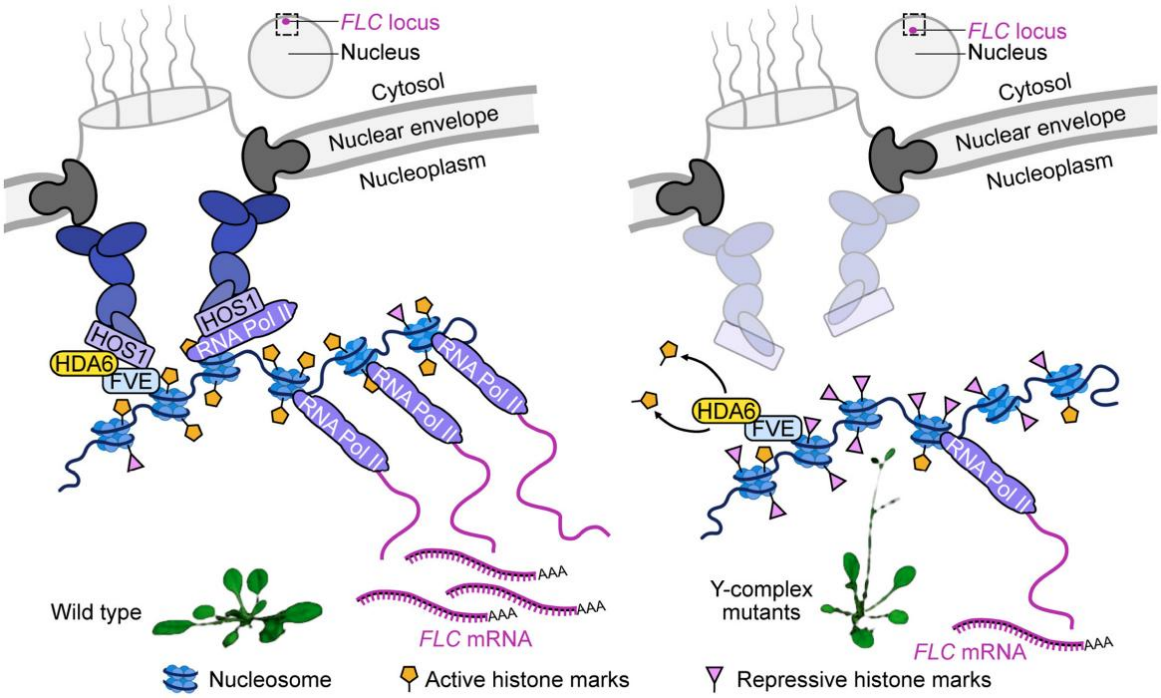
How a nuclear pore complex regulates *FLC*. Nup96 of the Y-complex interact with RNA Polymerase II and histones to bring the *FLC* locus physically closer to the edge of the nucleus, upregulating gene expression. Adapted from (Huang et al. 2023), Figure 8.

In *Arabidopsis*, the Y-complex is made of NUCLEOPORIN43 (Nup43), 85, 96, 107, 133, and 160 proteins, as well as SECRETORY13 (SEC13) and SEC13 HOMOLOG1 (SEH1) (Tamura et al. 2010). Further, HIGH EXPRESSION OF OSMOTICALLY RESPONSIVE GENES1 (HOS1) co-precipitates with Nup43, suggesting HOS1 functions in the Y-complex (Tamura et al. 2010). Curiously, several *nup* mutants flower earlier than the wild type, including *nup85-1*, *nup96-1*, *nup107-3*, *nup160-3*, and *hos1-3*. To unravel the underlying genetic intricacies, the authors conducted RNA-seq experiments with each mutant and found that these mutants displayed remarkably similar expression patterns of differentially expressed genes. Genes in common included the upregulation of the floral activating genes *FLOWERING LOCUS T (FT)* and *SUPPRESSOR OF OVEREXPRESSION OF CO 1* (*SOC1*) and the downregulation of the floral repressor gene *FLC*. Histone modification is a well-described mechanism for controlling gene expression. Therefore, the authors used ChIP-qPCR to determine the type of histone modifications within the *FLC* locus in the background of Y-complex mutants. Overall, the histone modifications lined up with the expression data: there were more repressing modifications on the histones in the *FLC* locus in the Y-complex mutants than in the wild type. While intriguing, these data did not explain how members of the Y-complex affect histone modifications and thus gene expression.

There are several prerequisites that must be met for a gene to be expressed. Briefly, DNA is wound around histones to form chromatin, and the histones must be modified such that DNA is accessible to RNA polymerase. HISTONE DEACETYLASE 6 (HDA6) is a protein that can deacetylate and methylate histones, thereby augmenting gene expression (Earley et al. 2006). Through a combination of bimolecular fluorescence complementation, co-immunoprecipitation, fluorescent in situ hybridization, and ChIP-qPCR, the authors confirmed that members of the Y-complex not only interact with HDA6 but also with RNA Polymerase II and the histones themselves. With these data, the authors present a compelling model whereby the *FLC* locus is brought closer to the edge of the nucleus, and this interaction upregulates *FLC* expression, ensuring that plants flower at the right time. Looking ahead, one cannot help but wonder what factors regulate *FLC*-Y-complex tethering, such as vernalization, photoperiod, circadian clock control, and stress resilience.

## Data availability

This manuscript does not have any data associated with it to make available.

## Dive Curated Terms

The following phenotypic, genotypic, and functional terms are of significance to the work described in this paper:

HDA6 Gramene: AT5G63110
HDA6 Araport: AT5G63110

FLC Gramene: AT5G10140
FLC Araport: AT5G10140
